# Evaluating the Dynamic Performance of Interfacial Pressure Sensors at a Simulated Body-Device Interface

**DOI:** 10.33137/cpoj.v4i1.36059

**Published:** 2021-05-19

**Authors:** M Hamilton, H Sivasambu, K Behdinan, J Andrysek

**Affiliations:** 1 Institute of Biomedical Engineering, Faculty of Applied Science and Engineering, University of Toronto, Toronto, Canada.; 2 Bloorview Research Institute, Holland Bloorview Kids Rehabilitation Hospital, Toronto, Canada.; 3 Department of Mechanical and Industrial Engineering, Faculty of Applied Science and Engineering, University of Toronto, Toronto, Canada.

**Keywords:** Calibration, Interface Pressure, Pressure Sensor, Pressure Measurement, Prosthetics, Repeatability, Sensor Evaluation

## Abstract

**BACKGROUND:**

Pressure sensing at the body-device interface can help assess the quality of fit and function of assistive devices during physical activities and movement such as walking and running. However, the dynamic performance of various pressure sensor configurations is not well established.

**OBJECTIVE(S):**

Two common commercially available thin-film pressure sensors were tested to determine the effects of clinically relevant setup configurations focusing on loading areas, interfacing elements (i.e. ‘puck’) and calibration methods.

**METHODOLOGY:**

Testing was performed using a customized universal testing machine to simulate dynamic, mobility relevant loads at the body-device interface. Sensor performance was evaluated by analyzing accuracy and hysteresis.

**FINDINGS:**

The results suggest that sensor calibration method has a significant effect on sensor performance although the difference is mitigated by using an elastomeric loading puck. Both sensors exhibited similar performance during dynamic testing that agree with accuracy and hysteresis values reported by manufacturers and in previous studies assessing mainly static and quasi-static conditions.

**CONCLUSION:**

These findings suggest that sensor performance under mobility relevant conditions may be adequately represented via static and quasi-testing testing. This is important since static testing is much easier to apply and reduces the burden on users to verify dynamic performance of sensors prior to clinical application. The authors also recommend using a load puck for dynamic testing conditions to achieve optimal performance.

## INTRODUCTION

Pressure sensing in mobility assistive technology (MAT) can inform the fitting of patient-customized devices such as prostheses and orthoses. For example, lower-limb prosthetic setup can take multiple weeks from the first to the final optimized fitting.^1^ Each fitting session features both static (i.e. standing) and dynamic (i.e. walking) weight-bearing assessments, where the clinician relies on their visual perception and fitting experience, as well as patient feedback to iteratively refine the fit and function of the device.^1^ Both static and dynamic assessments provide critical information, however dynamic assessments provide more pertinent information related to everyday mobility use.^2^ The integration of pressure-sensing into the MAT can help to quantify pressures at the MAT-body interface, affording clinicians more objective assessments, and thus improving overall fit and performance of the device.^3,4^

There are several commercially available interfacial pressure sensors, with varying technologies such as force-sensitive resistors, strain gauges, quantum tunneling composites, strain gauges and others.^5,6^ Most studies evaluating these sensors focus on static testing. The few sensor evaluation studies that have examined dynamic loading conditions, do not closely characterize the patterns of walking.^7–9^ Parmar et al. and Khodasevych et al. used 10 cycles of load application with 30 seconds on and 30 seconds off to simulate dynamic wear of a prosthesis.^6,10^ However, the typical walking cycles are much more dynamic repeating at approximately one Hertz (Hz) frequency.^11^ Hence, the mobility-relevant dynamic performance of interfacial pressure sensors remains to be established.

Aside from the loading patterns, previous research has shown that performance can greatly vary depending on the way a sensor is configured in its sensing application.^9,10^ The force-resistance relationship of a thin film sensor depends on factors such as sensor shape, geometry, and design, as well as the way by which the forces are applied to the sensor.^12^ In fact, sensor manufacturers recommend that sensors be tested and calibrated under conditions that closely match their application.^12^ However, this is difficult to do for MAT applications, due to the complex nature of the interface. For example sensor manufacturers recommend that the area of applied load be held constant at an area slightly smaller than the sensors’ sensing area.^12–14^ However, such conditions are not representative of the dynamic conditions of a body-device interface, where the loading area fluctuates and typically is larger than the sensing area. Sensor performance has been shown to vary with the area of applied load, however, testing has not been performed under dynamic conditions,^15^ despite well documented differences in sensor performance under static and dynamic conditions.^16^ Previous research with piezo-resistive sensors has reported a trade-off between the dynamic performance (hysteresis error) and the static sensitivity, as increased stiffness will alter the viscoelastic behavior causing hysteresis and reduce static sensitivity.^17–19^ Commercially available pressure mapping systems designed specifically for the MAT-body interface, such as the F-socket, utilize an array of sensors to provide pressure profiles over a large portion of the interface. However, these systems have limited clinical usage since they can be costly, bulky, cumbersome to apply, and suffer from performance issues including failure due to creasing; there is also a lack of information about their dynamic performance.^5,18^ As such, current understanding of dynamic performance of sensors in MAT-representative interfaces is limited, thus restricting the effective use of these sensors.

The overall objective of this study was to evaluate the effects of previously identified setup conditions (load area and presence of an elastomeric interface ‘puck’) on the dynamic performance of two common commercial pressure sensors. A sub-objective of the study was to understand the effects of two calibration techniques on sensor performance, including calibrating under matched-area (MA) and simplified, generalized-area (GA) conditions. As such, this study aimed to establish conditions and protocols that simulate dynamic testing of interfacial pressure sensors at the body-device interface, and empirically inform the use of the sensors for improved performance.

## METHODOLOGY

Testing was performed to assess the effect of area of applied load and sensor calibration method on sensor dynamic performance under two loading configurations: with and without a puck. The puck causes the force applied to the sensor to be concentrated over a particular area of the sensor.^12^

### SENSORS

This study was performed on two commercially available sensors, the QTC™ SP200-10 sensor (Peratech Ltd, Richmond, North Yorkshire, UK) and the ThruMode™ FSR (Sensitronics, Bow, WA, USA). These sensors were selected due to their high performance and broad use in other studies.^6,10^ Table 1 displays sensor specifications.

**Table 1:** Sensor model specifications

Parameter	QTC™ SP200-10	Half Inch ThruMode™ FSR
Manufacturer	Peratech Ltd.	Sensitronics Inc.
Sensing Diameter (mm)	10	12.7
Thickness (mm)	0.45	0.43
Claimed Operating Range (N)	0.1 to 20	0.26 to 26^a^
Single Part Repeatability (%)	N/A^b^	5
Part-to-Part Repeatability (%)	4.5	15

a Reported as 0.3 – 30 psi, converted to N using sensing area.

b Not reported.

### DATA ACQUISITION SYSTEM

Time and force data were collected using the Instron Bluehill Universal software. Resistance values were collected using a Keithley 6500 6 ½ Digit Multimeter (DMM) (Tektronix, Inc., Beaverton, OR, USA) and Tektronix’s proprietary KickStart software (Tektronix, Inc., Beaverton, OR, USA). All data were collected at 500 Hz. Resistance, force, and time data were analyzed using MATLAB v19 (The MathWorks, Inc., Natick, MA, USA).

### TESTING APPARATUS

An apparatus designed to simulate human tissue developed in a previous study evaluating pressure sensors under static conditions was used in this study.^15^ A 2 cm layer of soft translucent silicone (Renew^®^ Silicone 10, Renew^®^, Easton, PA, USA), shown to mimic behavior of human tissue,^20-22^ was placed over the Instron base platen. An Instron 5944 Universal Testing System with a 100 N load cell (Instron, Norwood, MA, USA) applied loads up to 10 N. This force range was selected as it is within both sensor’s working range and represents forces and pressures applied at the body-device interface in various biomedical applications.^23, 24^

Loading tip attachments (contact diameters of 5 mm, 8 mm, 15 mm, and 25 mm) were 3D-printed (PLA White Material; Ultimaker 2 Printer; Ultimaker B.V., Netherlands) and press-fit to the Instron’s upper compression platen. The 8 mm tip adhered to manufacturer recommendations for minimum coverage of FSR sensing area (i.e., load applicator 20% smaller than sensing diameter of sensor).^12^ This sizing prevented interactions with the spacer and adhesive surrounding the sensing area, while ensuring much of the sensing element was activated. The 5 mm tip represented conditions when a portion of the sensing area is loaded (e.g., point load). Clinically relevant conditions in which the loading area surpasses the sensing area are represented by the 15- and 25-mm diameter tips.

**Figure 1:**
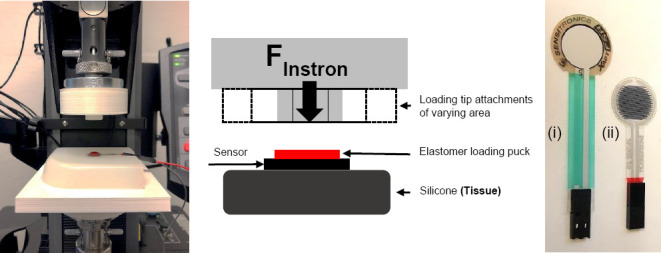
Actual setup, labelled schematic showing configuration with loading puck, and photo of sensors: (i) Sensitronics, (ii) Peratech

To understand the effects of sensor configuration, a loading puck was used as per sensor manufacturers’ recomm-endations under half of the conditions tested.^12^ A silicone loading puck (1.5 mm thickness, 8mm diameter, and durometer 60 shore a hardness) guaranteed the force was transferred entirely through the sensing area. The effect of the loading puck on the system’s phase and magnitude response was assumed to be negligible as the manufacturer recommends a silicone actuator to improve performance in cases of inconsistent force actuation;^12^ consistent responses with the loading puck were confirmed in pilot testing. Previous work indicated the use of the loading puck and omission of a rigid backing produced the best repeatability for both sensors.^15^ Figure 1 displays a photo of the setup and a labelled schematic indicating sensor configuration with the loading puck.

### PROTOCOL

#### Application Conditions

To evaluate the effects of load area and elastomer puck presence on sensor dynamic performance, a full factorial experiment was conducted using eight application conditions: four loading tip areas, both with and without an elastomer puck. The order of application conditions was randomized to minimize potential testing bias.

#### Sensor Conditioning

Prior to testing each sensor, manufacturer guidelines for sensor conditioning were followed,^13^ in which 110% of the maximum test load (11 N) was applied to the sensor for 30 seconds, and then removed for 30 seconds. This cycle was repeated four times.

#### Sensor Calibration

Prior to dynamic testing, a force sweep from 0 to 10 N was applied to the sensor at a loading rate of 0.67 N/s (i.e. loading duration of 15 s). This force sweep was repeated three times and the corresponding resistance and force data were curve-fit in MATLAB to characterize the sensor’s force-resistance curve for a given configuration (i.e. area and puck configuration).^25^ An exponential relationship (1) was selected to convert resistance output to pressure values for the subsequent tests based on manufacturer recommendations, literature, and best fit.^12,25^


(1) $$y=ax^{-b}$$

Two different calibration methods were used: MA and GA calibration techniques. Though sensor manufacturers recommend calibration conditions that imitate sensor use, this is not always possible at the body-device interface (i.e., inconsistent actuation and area of applied load); showcasing the importance of the simplified GA calibration method. The MA calibration is a more accurate, time-consuming method in which the exact configuration used during testing is matched during the calibration. The second method, GA calibration, is a time-saving approach where one configuration used during calibration is then applied to multiple configurations (i.e. different areas) during the experimental testing. For this study, both calibration methods were used to convert the same set of experimental data (sensor resistance) to force measurements, enabling a comparison between the used performance measures (i.e., Normalized root-mean squared error (NRMSE) and hysteresis error (HE)). Specifically, for the MA calibration, the calibration equation for each configuration was applied to the experimental data. For the GA calibration, only the calibration equation from the 8 mm puck was applied to each set of experimental data.

### SENSOR TESTING

#### Hysteresis Testing

Hysteresis is the difference in sensor output at the same force when the sensor is being loaded and unloaded and is commonly used to assess the performance of FSRs.^24,26^ To understand the sensor’s dynamic performance and identify hysteresis effects, the sensor was loaded from 0 to 10 N and then unloaded to 0 N at rates of 10 N/s (duration of two seconds). This was repeated 3 times. This loading rate was selected to analyze the hysteresis effects under conditions similar to dynamic loading: one second each of loading and unloading in the test is comparable to the average gait cycle time of approximately one second.^27^

#### Dynamic Testing

A square wave profile was applied to the sensor: loaded to 10 N, held for 1 second, unloaded to 0.5 N, held for 1 second, and repeated 10 times. The profile was intended to roughly approximate the weight bearing and non-weight bearing loading patterns during walking gait.

### ANALYSIS

Sensor performance was evaluated by analyzing accuracy and hysteresis. Researchers identified these performance measures during the evaluation of an interface force/pressure sensor.^10,28^ Accuracy errors, evaluated in both hysteresis and gait testing, was calculated using a normalized root-mean square error (NRMSE). The NRMSE, is calculated by dividing RMSE by applied force of 10 N and then converting the value to a percentage (2):


(2) $$NRMSE=\frac{RMSE}{\overset{-}{F}}*100\%$$

Hysteresis error (HE) was calculated by taking the maximum difference in sensor output (loading versus unloading) for a given force level. The hysteresis difference, ^F^unloading – ^F^loading, was calculated at each force from 0.5 to 10 N at increments of 0.1 N. The equation used to calculate HE (3):


(3) $$HE=\frac{F_{\text{unloading}}-F_{\text{loading}}}{\overset{-}{F}}*100\%$$

The hysteresis error was normalized by dividing by the maximum force of 10 N, and then converted to a percentage. For each trial, the hysteresis error was calculated at the force with the greatest hysteresis difference. An additional measure of sensor performance was the coefficient of variation (CV).^9,26^ Typically, clinical applications require a CV of less than 10%.^6^

An analysis of variance (ANOVA) was used to compare the effects of calibration method and puck on the NRMSE and HE for each sensor model (i.e., Peratech and Sensitronics). All main effects, 2-way and 3-way interactions were evaluated with p<0.05 indicating significance. Insignificant effects were then removed from the model, and significant main effects, 2 and 3-way interactions were reported. A paired t-test was performed on each set of results (i.e., NRMSE, HE) to quantify differences in sensor performance. JMP^®^ Pro 14 software was used (SAS Institute Inc., Cary, NC, USA).

## RESULTS

### DYNAMIC HYSTERESIS TESTING

**Figure 2:**
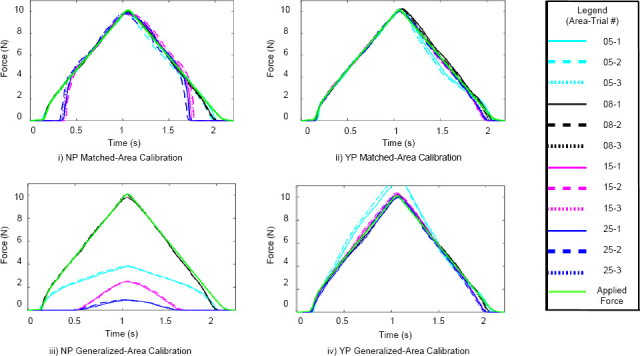
Force vs. time plots for two-second hysteresis tests for Peratech sensor using MA and GA calibration methods.

**Figure 3:**
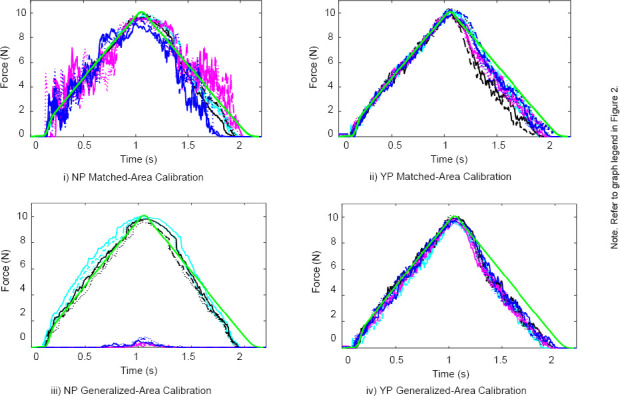
Force vs. time plots for two-second hysteresis tests for Sensitronic sensor using MA and GA calibration methods.

**Figure 4:**
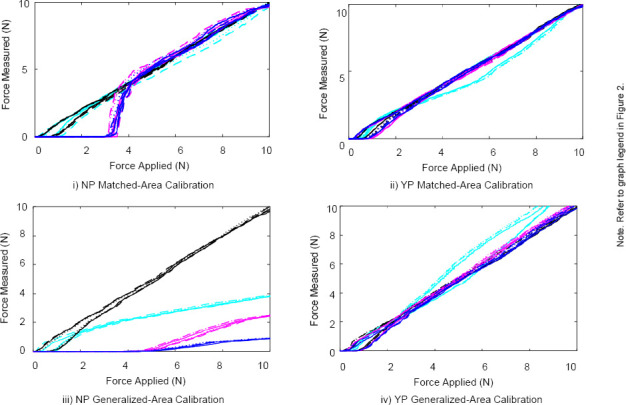
Force measured vs. force applied plots for two-second hysteresis tests for Peratech using MA and GA calibration methods.

**Figure 5:**
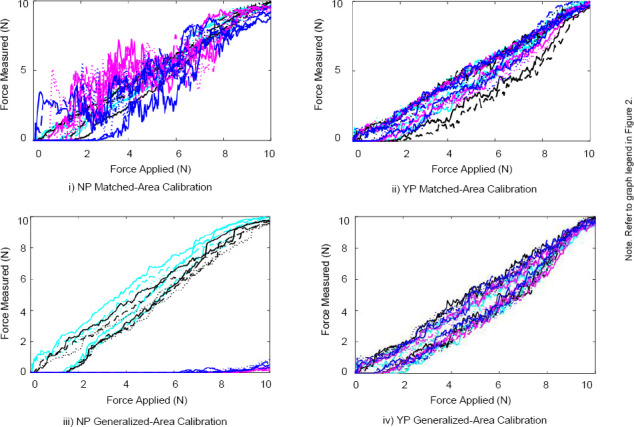
Force measured vs. force applied plots for two-second hysteresis tests for Sensitronics using MA and GA calibration methods.

The force applied versus time plots for the Peratech and Sensitronics sensors are displayed in Figure 2 and Figure 3. The force applied versus force measured representing the hysteresis curves for the Peratech and Sensitronics sensors are displayed in Figure 4 and Figure 5. Subplots are grouped by configuration: no elastomer puck (NP) and with elastomer puck (YP); as well as calibration method: MA and GA. Line colours distinguish the area of applied load, and line styles distinguish the trial number, as shown in the legend. NRMSE and HE values for the applications conditions are displayed in Table 2.

**Table 2:** Hysteresis and NRMSE results.

Config	Diameter (mm)	Peratech (%)				Sensitronics (%)			
		MA Calibration		GA Calibration		MA Calibration		GA Calibration	
		NRMSE	HE	NRMSE	HE	NRMSE	HE	NRMSE	HE
NP	5	2.6 ± 1.3	8.4 ± 1.7	32.8 ± 0.1	15.9 ± 4.6	5.4 ± 0.5	15.9 ± 1.4	7.8 ± 1.7	20.6 ± 2.8
	8	2.0 ± 0.2	8.6 ± 0.4	2.0 ± 0.2	8.6 ± 0.4	5.4 ± 2.1	16.0 ± 4.5	5.4 ± 2.1	16.0 ± 4.5
	15	11.1 ± 0.2	23.2 ± 15.3	46.8 ± 0.1	12.8 ± 3.9	9.9 ± 0.2	43.2 ± 8.6	57.3 ± 0.1	39.8 ± 14.6
	25	11.0 ± 0.1	12.2 ± 1.6	52.2 ± 0.3	11.5 ± 4.2	11.7 ± 2.1	48.3 ± 7.3	54.9 ± 0.3	38.6 ± 13.2
YP	5	4.8 ± 0.4	13.4 ± 0.8	9.2 ± 1.0	15.2 ± 0.6	5.3 ± 1.6	16.8 ± 2.9	9.9 ± 0.4	18.7 ± 2.7
	8	1.7 ± 0.4	7.8 ± 1.4	1.7 ± 0.4	7.8 ± 1.4	9.5 ± 0.9	25.4 ± 1.4	9.5 ± 0.9	25.4 ± 1.4
	15	2.5 ± 0.2	9.5 ± 0.4	2.8 ± 0.2	8.0 ± 0.1	7.3 ± 1.8	19.8 ± 2.5	9.8 ± 0.7	20.9 ± 1.9
	25	2.0 ± 0.3	8.8 ± 0.9	3.0 ± 0.1	6.6 ± 1.0	6.6 ± 2.2	19.0 ± 5.4	8.7 ± 1.1	22.4 ± 3.8

In Figure 2 and Figure 3, the applied force waveform is displayed in green on the plot, as indicated in the legend. These figures provide a visualization of the sensor’s accuracy in each configuration. Overall, the Peratech sensor exhibits higher accuracy than the Sensitronics sensor. The Sensitronics sensor signal exhibits more noise. The addition of the puck shows a significant improvement in sensor accuracy, especially in the GA calibration data. Without a puck, the GA calibration data accuracy reaches roughly 50% for the larger areas with both sensors. A dead band appears for the Peratech sensor in the NP condition with areas larger than the sensing area (15 and 25 mm), where no force is measured until approximately 3.5 N. In the MA calibration conditions, following the dead band, the data reaches 10 N because each data set was calibrated individually, with a different set of resistance values corresponding the force values for each configuration.

Overall, as seen in Figure 4 and Figure 5, the hysteresis error was significantly less for the Peratech sensor, compared to the Sensitronics sensor (p=0.01). For both sensors, the addition of the puck appeared to reduce hysteresis errors. The dead band described above can also be seen in these hysteresis plots. For both sensors, the NRMSE is below 10% for the majority of cases, with most exceptions occurring under conditions without a loading puck at the larger areas. While the HE is under 10% for most conditions for the Peratech sensor, all the HE values for the Sensitronics sensor are above 10% recommended for use in clinical applications.^6^

### DYNAMIC TESTING

NRMSE values for both sensors under the different application conditions are displayed in Table 3. With the MA calibration method, the NRMSE is below 10% for both sensors, meeting the desired threshold for accuracy in clinical applications.^6^ Using the GA calibration method with a puck, the CV across the areas averaged 8.8 ± 3.8 % for both sensors. Without a puck for the GA calibration method, CV values exceed 100% for the larger areas for both sensors.

**Table 3:** Dynamic testing - NRMSE

Configuration	Area	Peratech Sensor		Sensitronics Sensor	
		MA Calibration	GA Calibration	MA Calibration	GA Calibration
NP	05	3.7 ± 1.3	112.9 ± 2.8	8.9 ± 1.6	6.8 ± 0.9
	08	6.2 ± 0.3	6.2 ± 0.3	5.0 ± 0.8	5.0 ± 0.8
	15	6.6 ± 0.8	118.0 ± 5.9	8.4 ± 0.4	317.9 ± 18.3
	25	6.1 ± 0.2	558.3 ± 16.6	5.4 ± 0.6	185.6 ± 6.5
NP	05	4.8 ± 0.1	17.2 ± 0.0	4.2 ± 0.8	11.5 ± 1.3
	08	4.9 ± 0.6	4.9 ± 0.6	6.8 ± 0.4	6.8 ± 0.4
	15	3.7 ± 0.2	6.3 ± 0.3	5.9 ± 0.7	6.3 ± 0.8
	25	2.9 ± 0.1	6.8 ± 0.5	7.3 ± 0.1	10.8 ± 1.2

In the GA condition, similar trends showing the effects of the area can be seen in the hysteresis (Table 2) and dynamic (Table 3) results. In general, the NRMSE is lowest for GA with matched areas (8mm diameter loading tip and 8mm sensor area) and accuracy is decreased with higher NRMSE values for the smaller (5mm) diameter loading tip. This is most evident for the Peratech sensor. NRMSE is further increased for the two larger diameters (15 and 25mm).

## DISCUSSION

This study aimed to uniquely establish the methods and baseline data for relevant setup and calibration configurations and testing under dynamic loading patterns, for two commonly used pressure sensors. The study applied dynamic testing conditions that, compared to previous work, were much more representative of patterns present in human walking gait. The findings suggest that both sensors exhibited similar performance during dynamic testing that agree with accuracy and hysteresis values reported by manufacturers and in previous studies assessing mainly static and quasi-static conditions. In addition, the calibration method was found to significantly influence sensor performance, although much less so when an elastomeric loading puck was applied to the sensor.

Researchers have cited hysteresis and accuracy as key requirements of a successful interfacial sensor, although performance under dynamic conditions is often a limitation.^10,26^ Overall, the dynamic performance of the two commercial sensors is quite similar, however the Peratech generally exhibited better performance (i.e. higher accuracy) with lower hysteresis and dynamic testing errors.

The calibration method, as found in previous work, had a significant impact on the dynamic sensor performance.^15^ However, GA calibration can be used in place of MA calibration when using a loading puck to ensure the load is transmitted through the sensing area, regardless of the load applicator size. GA calibration is significantly easier and more practical to perform.

The NRMSE values reported in this study agree with the accuracy values reported in the study by Parmar et al^6^ ranging from 94.8 to 96.0% (equivalent to an error of 4.0 to 5.2%) for the Peratech sensor and 90.8 to 94.0% (equivalent to an error of 6.0 to 9.2%) for the Sensitronics sensor. These values are within the range of the errors seen for the 8 mm loading tip applicator conditions using MA calibration. Similarly, the hysteresis error observed for the Peratech sensor with the 8 mm loading tip applicator conditions using MA calibration agrees with the manufacturer reported hysteresis error of 8.5%.^29^ Finally, the CV of 8.8 ± 3.8% for dynamic testing with the loading puck agrees with previous works performing static testing under the same conditions (7.6 ± 3.6%).^15^ These findings provide new evidence suggesting that the sensor performance is not adversely affected by dynamic loading that is at the frequencies relevant to mobility. It further suggests that the performance is sufficient for most clinical applications.^6^

Previous studies have suggested potential performance trade-offs between static and dynamic performance,^17,18,30^ however for the sensors and conditions tested here this did not appear to be the case. In part this may be because the loading frequencies associated with gait mobility are not high enough to adversely affect dynamic performance. Alternatively, the findings may be influenced by the type of sensor technology or material used. Nevertheless, for gait related MAT applications the findings have important implications on the utilization of the thin film sensors, suggesting that static testing and calibration as suggested by manufacturers might be adequately sufficient, without the need for further dynamic testing. This is important, since whereas static testing can simply be performed with a dead weight, dynamic testing requires specialized equipment that is not readily accessible. Implementation of objective measures produced by pressure sensors and similar systems are typically confined to research settings due to their cost and lack of portability.^31,32^ However, the potential simplification of testing protocols and assurance of their relevance to dynamic testing, is important for feasibility in the case of the use of these sensors in research as well as clinical realms.

A limitation to the study is the assumption of uniformity in tissue loading. Testing performed included a uniform layer of silicone simulating tissue at the body-device interface. In real life, the anatomy of a limb includes inconsistencies in tissue properties and bony prominences that can affect compliance and curvature. Additionally, effects of frictional shear forces, temperature, and curvature were not examined in this work, but have been shown to affect pressure measurements. Additionally, it is possible that a portion of the measured errors may be associated with the dynamic response of the testing machine setup, rather than sensor performance. The limits of the sensors should also be explored in dynamic applications, including higher cycle frequencies. A faster cycle time, (i.e. 1 second instead of 2 seconds) would be more representative of the dynamics of normal gait. Finally, the feasibility of incorporating a loading puck in a body-device interface or in an actual clinical application was not assessed here. Future work will need to consider feasibility and performance of adding pucks, including their design (i.e. thickness, hardness) and effects on aspects such as comfort and application time.

## CONCLUSION

This study develops and tests a unique protocol for the dynamic testing of pressure sensors at the body-device interface, and addresses concerns with existing approaches including applicator size, loading profile, and sensor conditioning and calibration. Overall, when using sensor configurations recommended by manufacturers both sensors exhibited performance sufficient for use in clinical applications. The foundational knowledge established by this work reveals that existing thin film pressure sensors may be a suitable tool for measuring pressures at the body device interface, such as prosthetic sockets and orthosis, and can do so for dynamic conditions such as gait. Future work in this area should examine the effects of additional properties unique to the body-device interface.

## DECLARATION OF CONFLICTING INTERESTS

The authors declare that they have no competing interests.

## AUTHOR CONTRIBUTION

**Megan Hamilton:** contributed to the study concept and design, data gathering, analysis and interpretation, and contributed to the drafting of the manuscript.

**Harry Sivasambu:** contributed to data analysis and interpretation, drafting of the manuscript, and read and approved the final manuscript.

**Kamran Behdinan:** contributed to the study concept and design, contributed to the drafting of the manuscript, and read and approved the final manuscript.

**Jan Andrysek:** contributed to the study concept and design, analyzed and interpreted data, contributed to the drafting of the manuscript and read and approved the final manuscript.

## SOURCES OF SUPPORT

This work was supported by the EMHSeed program from the University of Toronto.

## ETHICAL APPROVAL

Ethical approval was not needed for this study.
